# Focus Group Findings to Support the Preliminary Development of the Augmented Reality Education Experience (AREduX)

**DOI:** 10.7759/cureus.26304

**Published:** 2022-06-24

**Authors:** Eva Peisachovich, Bill Kapralos, Celina Da Silva, Adam Dubrowski, Naida L Graham, Regina Jokel

**Affiliations:** 1 Medical Education and Simulation, York University, Toronto, CAN; 2 Information Technology, Health Education Technology Research Unit, Ontario Institute of Technology, Oshawa, CAN; 3 Faculty of Health Sciences, Ontario Tech University, Oshawa, CAN; 4 Health, York University, Toronto, CAN; 5 Speech-Language Pathology, Temerty Faculty of Medicine, University of Toronto, Toronto, CAN; 6 Speech-Language Pathology, Rotman Research Institute, Baycrest Health Sciences, Toronto, CAN

**Keywords:** education, simulation, healthcare providers, dementia, augmented reality

## Abstract

Dementia is considered a global health priority with projections of the disease set to increase dramatically across the world. Current support for persons living with dementia (PLWD) relies on long-term care and local service centers to provide education and support. Augmented reality-based programs continue to gain momentum across health sectors, becoming an innovative approach that provides an opportunity to have a visceral experience, which can deepen understanding and provide an embodied perspective of other groups within a relatively short time frame. There is increasing interest in developing approaches to aid patient care outcomes for PLWD and their caregivers. Hence, healthcare providers (HCPs) who are appropriately trained and equipped to provide quality care to PLWD are essential and of international concern. The purpose of this research program is to develop an augmented reality (AR) education experience (AREduX), a proof of concept prototype in the form of a digital resource that uses AR to simulate the physical and cognitive symptoms that PLWD experience. The findings from a stakeholder focus group will allow for the preliminary development of the AREduX.

## Introduction

Dementia is a devastating disorder for sufferers and their families, and it imposes a substantial burden on caregivers, healthcare providers (HCPs) and the health care system. The estimated cost to the Canadian economy of caring for persons living with dementia (PLWD) is $10.4 billion per year, and rising [1]. Family caregivers and HCPs play a critical role in providing care for PLWD. Research has shown that greater empathy on the part of HCPs leads to better care and improved patient satisfaction and outcomes [2,3]. Moreover, there is a growing body of evidence within the healthcare community suggesting that the development of compassion can profoundly affect physical and psychological health [2,4-6]. Thus, it is essential that the training of HCPs incorporates methods that will promote greater empathy for and understanding of the experiences of PLWD.

To date, there are limited programs that train HCPs or caregivers to develop empathy [7]. Research indicates the variable impact of training on a range of staff outcomes, yet there is little evidence about the most effective approaches to the design, delivery and implementation of training to prepare HCPs caring for PLWD [8]. Currently there is limited research within the field of augmented reality (AR). To our knowledge there are no studies aligned with the care of PLWD, nor its application as an educational training medium to teach empathy. Since AR signifies a variation of virtual reality (VR) and plays a supplemental role rather than a replacement of reality [9], a scoping review which examined VR as an intervention in dementia care education, including HCPs and caregivers of PLWD, found that there are existing indications that immersive technologies (VR, AR) may be an effective intervention to train HCPs and caregivers to be more empathetic, to understand symptoms and respond effectively, and provide evidence on the positive impact of immersive simulation technology [10]. Although there are scientific efforts to evaluate the use and impact of immersive technologies in dementia care education, it has not yet been evaluated in an appropriate way, since studies are rare and do not address effectiveness [10]. It is recognized that AR in dementia care education is a specific setting where students, HCPs, and caregivers must be prepared to interact with PLWD in a person-centered and sensitized manner [11,12]. The findings from our proposed research program will substantially contribute to guiding further research aligned with implementing AR as an experiential education and training element to enhance empathy among HCPs and caregivers of PLWD and provide important knowledge about how AR impacts the awareness of HCPs and caregivers about how dementia impacts how PLWD navigate their world.

Simulation in medical education is a well-established pedagogical practice [13]. It provides a viable alternative to practice with actual patients, allowing medical trainees the opportunity to train until they reach a speciﬁc competency level. Simulation ranges from de-contextualized bench models and computer-generated (virtual) environments to high-fidelity recreations of actual operating rooms [14]. One of the prevailing arguments for using simulation in the learning process of trainees is the ability to engage the trainee in the active accumulation of knowledge by doing with deliberate practice. At the same time, it also allows for careful matching of the complexity of the learning encounter to the trainees’ current level of advancement [15]. The increase in technological advancements over the last couple of decades has led to the availability of affordable computation power. This, in addition to the shrinking of electronic components, and the rise in affordable consumer-level hardware, mobile computing platforms (e.g., tablets, laptops, and smartphones) have seen the application of emerging technologies including VR and AR, across many areas of education including medical simulation. VR has been defined as “the use of computer modelling and simulation that enables a person to interact with an artificial three-dimensional (3-D) visual or other sensory environments” [16]. The user/trainee is completely enveloped in the virtual (artificial) world, typically through a head-mounted display (HMD) worn by the user.

In contrast, in an AR environment, digital information overlays the physical surroundings leading to an environment that is both real and virtual [17]. This is achieved using an HMD worn by the user or via a tablet-type device (e.g., smartphone), whereby the digital (virtual) object is generated and surrounded by the real environment [18]. This combination of physical and virtual information allows AR to enhance further the well-established procedural simulation methods [19]. As Tang et al. describe, despite the availability of VR/AR technologies for several decades, the recent advances in visual technologies (along with computational improvements), have led to the development of new AR applications and drawn much attention to the field [20]. AR promotes better long-term memory retention and critical thinking, problem-solving, and communication skills development within an educational setting than traditional education methods [21]. More specific to medical education, AR-based applications have been applied across every stage of medical training where it has been suggested that AR may “foreshadow a new paradigm in medical education” [20]. As Dhar et al. described, within medical training, AR offers a safe learning environment that can enhance the learners’ experience thus providing a great potential to effectively and efficiently prepare medical professionals for the real world of practice [18]. A complete discussion of AR within the context of medical training is beyond the scope of this article, but an overview is provided by Dhar et al. [18] and Tang et al. [20].

This report describes the first phase of a project with the eventual goal of developing innovative approaches to enhance the education and training of HCPs working with PLWD, with a particular emphasis on improving empathy. To accomplish this goal, we plan to develop a prototype of an AR education experience (AREduX) that will offer experiential learning that simulates some of the effects of dementia and challenges experienced by PLWD. The immersive experience will be used for training, to give HCPs and caregivers of PLWD a sense of what it is like to live with dementia, to make them more empathetic and, therefore, more likely to deliver compassionate care.

In this report, we describe the preliminary stage in developing the AREduX, which involved consultation with relevant stakeholders using a focus group. The goal was to obtain caregivers’ and HCPs’ opinions and knowledge. We expected that their input would provide insight into, and understanding of, the lived experiences of PLWD and their caregivers. This insight will guide the design of the digital educational resource, and inform the elements that will be incorporated.

## Materials and methods

Focus group 

The focus group session was held in December 2021. Due to the ongoing COVID-19 pandemic, it was not possible to hold the focus group in person as initially planned and the focus group session was therefore held virtually via the Zoom video conferencing software (Zoom Video Communications, Inc. Santa Clara, CA). Conducting the focus group virtually was advantageous for the caregivers because they could participate without arranging alternative care for their family members with dementia. The Zoom software was also used to transcribe the audio resulting from the session. The focus group session was facilitated by a research assistant working on the project. To ensure that the facilitator was not distracted by any technical issues, another research team member assisted with hosting and recording the Zoom meeting. The transcription was subsequently reviewed by the team member who had assisted with the meeting, and errors in the automated transcription were corrected. The duration of the focus group discussion was 95 minutes.

Most of the questions included in the script were targeted at particular subgroups of participants. To ensure that input was obtained from everyone, if a participant from the group targeted by a question did not volunteer a response, they were asked if they had anything they would like to add. The facilitator used a script which is provided in Table 1. 

**Table 1:** Focus group questions

What role do you have: HCP, Caregiver of PLWD, PLWD, Instructional designer, Computer science expert, or Software developer?
For HCP/Caregiver:
In your opinion, tell me what makes a good HCP or caregiver?
What are your work-related goals when caring for PLWD?
What is the biggest challenge for you when caring for PLWD? What have you done about it or what are you going to do about it?
Tell me something positive about working with PLWD.
What’s the most interesting thing about your work with PLWD that we don’t know?
What personality trait of yours do you think is most valuable when caring for PLWD?
Tell me about a time you care for a PLWD. What part of the experience was most meaningful to you?
Tell me about a difficult client living with dementia, what made it difficult to work with them and what did you do to help them?
For PLWD:
What’s the best way your HCP or caregiver can take care of you?
What are some of the challenges you face every day?
What are the moods you typically experience during the day?
Do you have any concerns or worries in your day-to-day life?
Tell me something positive your HCP or caregiver did for you? Tell me something negative? How did it make you feel?
Have you come across a HCP or caregiver that was not patient with you?
Tell me about a difficult day you had with your HCP or caregiver? Did they resolve the issues? If not, how did you feel? What did you do?
For Other
What are ways we can integrate AR into long term care settings?
What is your experience with creating an AR experience to support learning?
What is your experience working with PLWD?
What is your experience with teaching learning contexts using AR?
Wrap-up questions:
What was the one thing you would like to be implemented into the development of the AREduX?
What are the most important things you've found out about AR during your learning or professional context?

Participants

A total of 12 participants joined the focus group. A range of stakeholders was included among the participants ensuring various perspectives, but they broadly represented two subgroups. The first subgroup (n=7) comprised participants with experience living with dementia or caring for PLWD, while the second subgroup comprised educational developers and experts in computer science. All participants met the following inclusion criteria: able to understand, read and speak basic English; willing to participate in a focus group; able to access the internet.

Recruitment process 

Participants who had experience with PLWD were recruited through Baycrest, an academic health sciences centre providing a continuum of care for older adults. This subgroup included one HCP, four caregivers, and two PLWDs. HCPs were recruited by word-of-mouth and fliers posted at Baycrest, which detailed the study. Caregivers and PLWD were recruited via the Baycrest Client Registry.

HCPs were individuals who care for PLWD in some professional capacity. Caregivers were carers for a person with any type of dementia who did not necessarily live with the person for whom they care. PLWD had been diagnosed with any type of dementia. We aimed to recruit PLWD who had scores from 18 to 25 (out of 30) on the Montreal Cognitive Assessment [22]. This range would identify participants with mild dementia who should be capable of answering questions; however, since current MoCA scores were not always available, participants with dementia may not have been capable of achieving scores in this range at the time of the focus group.

The second subgroup of participants (n=5) was recruited through purposive sampling and included two educational developers, two game development students, and one participant who was a software developer and research assistant.

Focus group challenges

There were some challenges with recruitment. The focus group was intended to be larger, but one HCP, four caregivers, and two PLWD agreed to participate and then withdrew shortly before the focus group started, or failed to show up. The high drop-out rate may have been due to caregivers being under much pressure due to the demands of caregiving. Recruitment of HCPs was made difficult by the COVID-19 pandemic. When the focus group was held, HCPs were overworked and seemed to have little spare time to participate in research. Despite these difficulties with recruitment, there were sufficient participants to enable us to obtain reliable and fulsome information.

In addition, the two participants with dementia were limited by their disease in their ability to contribute to the discussion. One had aphasia with empty speech and obvious word-finding difficulty, and when she spoke her intended message was often unclear. The other participant with dementia had fluent speech but frequently did not answer the question asked and instead spoke off-topic. Nevertheless, both participants with dementia were able to make some relevant and valuable points. The observed difficulty with communication enhanced our understanding of the experiences of PLWD and will contribute to our prototype development. However, the questions did provoke discussion which was relevant and informative for our purposes.

Some minor but unexpected events led to reduced participation from three participants. The HCP chose to participate while at work and appeared to experience numerous interruptions in the latter half of the session, hence participating only in the first half. Furthermore, one of the student game development participants had to leave before the session ended. Finally, the participant who was a software developer and research assistant contributed little and said she was just there to listen.

## Results

Qualitative analysis of results

The focus group transcription was qualitatively coded on a sentence-by-sentence basis using an open coding process that was not based upon a pre-existing theory. The coding was done using the NVivo qualitative data analysis software (QSR International, Doncaster, Australia), which facilitates the analysis of unstructured text such as focus group results. The software provides tools for classifying and sorting data, leading themes or patterns to become apparent. In the present analysis, categories of information (or nodes) were developed as part of the open coding process. Next, the relationships between the codes were examined and connections were made between them where relevant. Ultimately,

following an iterative process of refining the codes and their interconnections, we were able to identify central themes within the data. These themes are detailed below and are listed in Table 2, together with select quotations from participants.

**Table 2:** Themes and select participant quotations identified through qualitative data analysis PLWD: persons living with dementia; PSW: personal support workers

	Theme	Sub-theme	Representative quotations
1	Hurdles and challenges in caregiving		
		Relentless nature of caregiving	"Basically it's very, very hard, because you're there 24/7"
			"It's all night long, you're getting up all night long"
			"It's a constant, it never stops"
			"That is just so exhausting for people"
		Problems communicating	"I can't talk to him, I can’t understand what he's saying and I can't hear him, and nor can anybody else"
			"If she needs something it is very difficult to explain, like it’s a guessing game"
			"It’s very hard for her to express herself"
		Others don't understand how hard it is	"I don't think anybody can appreciate until you go through what we're going through, how very difficult it can be"
			"The more that people can understand what it really means to be 24 seven"
			"It would be really helpful if somebody were to be aware of what's going on"
			"If people really knew how much it hurt that they just abandon us alone"
2	Help from others (or lack of it)		
		Professional help	"They're very good at what they do and they're very fast"
			"Sending in a PSW for 15 minutes, to bathe him. It doesn't begin to cut what I really need"
			"If we have time, do them extra, and I do it for my clients to just give them a little bit relief"
		Lack of help	"But when I’m there alone and it's the middle of the night, it's just me"
			"The things that I’m doing I’m doing all by myself"
			"You're the only one there"
		Lack of family support	"Our kids don't understand what it is, they have no idea what it's like"
			"They would never believe how much it hurts. I feel completely abandoned by the kids"
3	Description of PLWD		
		Capabilities – tasks and activities PLWD needs help with	"You have to do the bath"
			"He's not understanding what he's not able to do"
			"He can't follow my directions"
			"To remind them to eat"
		Capabilities – tasks and activities PLWD can do	"My husband would say thank-you a lot"
			"She never forgot how to kiss"
			"She was taking Wheel-Trans on her own. People were meeting her at the other end"
			"She loves playing coffee on Lumosity"
		Dementia signs and symptoms	"Forgetting what day it is"
			"Loss of speech is part of the . . ."
			"She tends to think of herself only"
			"She starts breathing like she's running a marathon. And it's all part of the anxiety, and for no reason"
		Non-dementia signs and symptoms	"He’d get very tired every day"
			"She was so tired, staying in bed all the time"
			"I don’t hear too well"
			"He'd start the first few words. The first few words would be loud, but then the voice fell off"
			"It is a challenge for her to walk on the lawn"
			"It's the pain that is so hard to take"
4	Goals in caregiving		
		Safety	"Safety is really, really important"
			"Keep their environment as safe as we can possibly make it for them"
			"All of these things are safety things, it's so so important"
			"You gotta keep them safe, I mean that's one of the most important things that we do is how do we keep them safe"
		Keep PLWD happy, comfortable stimulated	"And stimulation, I’m trying to get her to try a day program or online activities"
			"Make them feel comfortable"
		Encourage independence	"Keeping them independent as much as we possibly can"
			"While she can still do certain things I insist that she does it"
			"Allow them to control as much as they can reasonably control. That's very important for them to have control"
			"I’ve been trying to force her to keep doing as much as possible, but is that right or is that wrong I don't know"
		Keep a routine	"Routine is very important for her"
			"Keep a routine"
5	Strategies and things that help with caregiving		
		Patience	"It takes a lot of patience"
			"I’m going to echo that too with patience"
			"The patience was the first word that came to my mind as well"
		Other essential carer characteristics	"Caring is the most important thing"
			"Try to have empathy for the person you're caring for, trying to understand what they're going through"
			"Putting yourself in someone's feet and thinking about them, what they are going through, and then, if you think that, and then you try to help them it is much different"
		Adapt to the PLWD's needs	"Not going by the book all the time"
			"It's not your agenda anymore, it's their agenda"
			"Allow them to control as much as they can reasonably control"
		Differentiate person and disease	"It's just understanding the disease process. It's not them, it's something else that is doing to them so that's why all this is happening"
		Other strategies	"Unless it is not related to the safety so then just distract them"
			"Make sure that every step is reinforced all the way, just reinforce as if it's a child or a dog"
			"And I would simply put my arms around him and tell him that everything was okay, and that will help to quiet him down"
			"Eventually, we put him on some pills and help to decrease the frustration and the anxiety"
		Self-care	"You also have to take time for yourself"
			"If you get burnt out you're no good to anybody"
6	Technology and equipment		
		Helpful	"When I lost my wife that was probably, one of the worst things I’ve ever felt my life, so when I got that monitor everything changed"
			"The big thing for me was these life alarm items. SOS button and GPS tracker, I actually got it"
			"Wearing the proper shoes, so that you don't fall, or you don't slip"
			"I use the cane only and I’m very happy with the cane"
			"My hearing aid"
			"I watched my wife on Lumosity and . . . she adores it"
		Unhelpful	"What do you do when I buy him an elevated toilet seat, but he won't sit down"
			"I bought him a chairlift because we have one set of stairs, but you know he began to be afraid to sit in the chair"
			"When I had a walker, I thought I would kill myself, I found that so dangerous"
			"She doesn't use a cell phone at all"
			"I tried Siri with my wife. It just doesn't work, really doesn't work for us"
		Technology caregivers would like to be created	"Anything that I could do to make it easier for me to hear him"
			"An interactive program where she can express certain things, and then the computer would be able to, she can draw back from the computer certain things that she forgot about"
			"For argument's sake, pictures of people with their names and she would forget these names, but then she can go back to the computer and the computer can guide her"
			"I can see a real use for that you know, just by having that hazard sort of light up"
7	AR design recommendations		
		Technology that could be developed	"If we're thinking just in an AR space, being able to label and actively amplify audio"
			"On a small little device, you can touch a button and have it amplify a certain phrase"
			"AR could provide purpose, fulfillment, and engagement to certain individuals"
			"Providing a level of companionship to people who might be lonely a lot of the time"
			"Being able to sense the emotions and neuro stimuli that occurs when certain activities are being undertaken and being able to capitalize on that and help give those individuals, a higher quality of life is really the challenge"
		Design advice	"For anyone designing these tools, you know, making it simple but also being able to address those needs"
			"The onboarding experience is really important because sometimes the people who use these devices may not be completely familiar with using technology"
			"Having to get a lot of people to test and look at it, and even those that are not considered like the people that would be using it . . . just so that you can find out how it would be interacted with constantly is something that's really important"
			"Because I don't play video games, so what most young people who have described this all to me, for example, assume I know how to use a controller to play a video game and I don't"

Qualitative findings 

There were seven themes generated via the qualitative coding, and these are elaborated upon in this section. The first six themes relate to experiences associated with dementia, while the seventh theme arose from a discussion of experiences with AR.

*Theme 1*: *Hurdles and Challenges in Caregiving*

This theme included any mention of something which increases the difficulty of caregiving. Elements of this theme were mentioned by all of the caregivers and by the HCP. In particular, they described the relentlessness of caregiving, and the three caregivers who live (or lived) with a PLWD emphasized this strongly. They noted how fatiguing caregiving is because it is perpetual, and happens around the clock.

Another significant challenge in caregiving was noted to be problems with communication, mainly comprising difficulty understanding what is said or meant by the PLWD. Two of the caregivers described particular problems in this regard. In one case, the PLWD had aphasia with word-finding difficulty and was mostly unable to generate meaningful speech. In the other case, the PLWD suffered from dementia associated with Parkinson’s Disease (PD), for which hypophonic speech is an established feature; as a result, this person was not able to speak, loudly enough to be heard, and this was a substantial barrier to communication that was frustrating and difficult for the caregiver. 

Another area that seemed to particularly add to the challenge is the fact that other people don’t understand the inherent difficulty of caregiving. The lack of acknowledgment and understanding from others was stressed by all of the caregivers. This relates to their perceptions about lack of support, which is further addressed as part of Theme 2.

Other difficulties included how hard it is to accept the situation, the fact that the PLWD can no longer do things that they used to, and that the PLWD is different from how they used to be.

*Theme 2*: *Help From Others (or Lack of it)*

This theme refers to any acknowledgment of professional or other (mostly family) help with caring for a PLWD. Caregivers were generally appreciative of professional support and particularly liked Personal Support Workers (PSWs). The caregivers seemed to agree, however, that the help they receive is insufficient and that ultimately they shoulder the responsibility and burden of caregiving all on their own. Lack of help from other family members seemed to be a sore point for caregivers and was mentioned by the HCP as well.

*Theme 3*: *Description of PLWD*

This theme included a description of the capabilities of the PLWD (activities they can and cannot do and perform), as well as signs and symptoms of dementia and of co-occurring morbidities. Tasks and activities the PLWD can no longer do at all, or can no longer do without help, including driving, traveling alone, bathing, grooming, feeding, getting into bed, taking others into consideration, remembering to do things, and following directions. Participants did mention things the PLWD is still capable of doing, but these seemed rather limited (e.g., saying thank-you, smiling). Things that the PLWD can no longer do outnumbered the things they have retained the ability to do. 

The signs and symptoms of dementia that were mentioned by the caregivers and the HCP included problems with memory (which seemed specifically to involve episodic memory), problems with speaking and understanding, and loss of the ability to take another’s perspective (resulting in selfishness and not taking their caregiver’s needs into account). Anxiety was also reported, and this is a common concomitant of dementia. 

The caregivers’ comments indicated that the people they care for are suffering from comorbidities. This was reflected in comments describing non-dementia signs and symptoms, such as fatigue, hearing loss, inability to speak loudly enough to be heard, difficulty walking, and pain. For two of the caregivers, these additional problems seemed to substantially increase their burden. In one case, the PLWD had PD, and the concomitant quiet speech, fatigue, and difficulty walking added considerably to the workload, while in another case there was a significant problem with pain as well as difficulty walking.

*Theme 4*: *Goals in Caregiving*

This theme refers to anything caregivers or the HCP aim to do which will benefit the PLWD for whom they care. They all seemed highly motivated to do their best in caregiving, and this theme comprises the things they do to help their PLWD, as well as their stated objectives for helping. Safety was a primary objective and was mentioned by all four caregivers and the HCP, with particular emphasis on keeping the environment safe. Strategies used to safeguard PLWD included arranging for a medical alert system so the PLWD can summon help, not allowing the PLWD to go out on their own, ensuring extra care is taken when bathing, and setting-up handrails, and ensuring the floor is kept clear of trip hazards. Keeping the PLWD happy, comfortable, and stimulated also seemed to be an important goal for these participants, as well as encouraging independence as much as possible, and keeping a routine.

*Theme 5*: *Strategies and Other Things That Help With Caregiving*

This theme comprises caregiver characteristics and strategies which were described as necessary or beneficial for caregiving. The most frequently mentioned requirement in a caregiver was patience. Empathy and caring were also perceived to be important, as was the ability of the caregiver to adapt to the PLWD’s needs. 

The caregivers and HCP mentioned a range of useful strategies. One that was mentioned frequently was the differentiation between the person and the disease; the idea is that it is easier to cope when the difficulties are blamed on the disease rather than the PLWD. Other useful strategies included distraction, reassurance, acknowledgment of the feelings of the PLWD, and in one case, medication. Caregivers also mentioned that it is important that they look after themselves to ensure that they remain able to care for their family members with dementia.

*Theme 6*: *Technology and Equipment*

This theme includes any mention of technology and equipment, whether helpful or not, including technology that participants would like to see developed. Equipment that was noted to be helpful included monitoring devices, such as a GPS tracker, a medical alert system, and an emergency call button, as well as basic equipment such as proper shoes, a cane, and a hearing aid. Helpful technology was mentioned by only one (caregiver) participant who noted that the PLWD for whom he cares can be kept occupied by, and obtains significant enjoyment from, playing Lumosity which is proprietary “brain-training” software.

Other equipment was tried but not found to be useful. Participants often noted that the PLWD was incapable of using devices that were intended to help. The person who cared for an individual who had both dementia and PD was very vocal about how frustrated she was that the person for whom she cared was unable or unwilling to use various devices which she purchased (e.g., stair lift, raised toilet seat). One of the participants with dementia noted that she was unable to use a walker and found it dangerous, although she found a cane helpful. The only technology that was mentioned in this regard was that one participant had tried using Siri (a voice-controlled personal assistant) with his wife (he didn’t specify for what purpose), but it wasn’t helpful. 

The caregiver participants had several suggestions for technology that they would like to see developed. Some of the items on this “wish list” were also discussed by participants who are educational developers or computer science experts (see Theme 7). Caregivers expressed a need for technology that would enable a PLWD to better communicate, either by making quiet speech audible or by helping with word-finding and/or generating a message. Other potentially helpful technology would be something that could calm or reassure a distressed PLWD who is on their own, something that would light up when a hazard is present, such as a tripping hazard, and something to keep the PLWD safe if they go near a stove.

*Theme 7*: *AR Design Recommendations*

This theme comprises a discussion about experiences with AR and about ways AR could be incorporated into Long Term Care settings. In contrast with the above themes, most of what was said on this topic were contributed by the educational developers and computer science experts. At the time that AR was discussed, these participants had already listened to the caregivers’ ideas. 

The suggestions made by the educational developers and computer science experts included ideas for technology that could be developed within an AR framework, as well as potentially useful design features. Suggestions for technology that could be developed in the future included a device to label and actively amplify audio, as well as using AR to develop something to provide fulfillment, engagement, and even companionship for PLWD. In addition, it was suggested that technology capable of sensing the emotions and reactions of a PLWD could ultimately be used to help PLWD. Guidance for the development of such technology was offered. In particular, it was felt that the user interface should be kept simple, and be thoroughly tested.

A summary of the themes is illustrated in Table 2. 

## Discussion

A focus group meeting was held to obtain stakeholders’ inputs to inform the development of an AREduX prototype which will ultimately be used to increase empathy for PLWD, among HCPs and caregivers of PLWD. We were able to obtain inputs from various stakeholders with various perspectives, which provided insight into the lived experiences of PLWD and caregivers. 

Caregivers described their struggles with caregiving. They find it relentless and exhausting, and they feel that they receive insufficient help or acknowledgment from others. Nevertheless, they were clearly motivated to do the best they could. 

Problems experienced by PLWD were also described. PLWD may experience difficulty undertaking simple tasks such as grooming, feeding, and getting into bed. They also suffer from memory loss and problems with communication, both in language production and comprehension. The communication problems seemed to present more of a hurdle in caring than did the memory loss. The PLWD who were described in the focus group suffered from comorbidities that caused symptoms in addition to dementia, such as hearing loss, mobility issues, hypophonic speech, and pain. 

Some of the elements mentioned in the focus group could be incorporated into the AREduX. Since the aim of the AREduX is to create a digital resource in the form of AR and expose users to an experience that gives them a sense of what it is like to live with dementia using the latest AR game development technology, our team will create a unique experience that simulates the effects of dementia for training purposes with the goal of making users more empathetic. The experience itself is intended to draw the user into the imagined world of PLWD.

The educational developers and computer science experts had little to no experience with AR in a health care setting. This lack of experience indicates that our project is valid and demonstrates a need for more work in this area. Further, the findings from data collected through the focus group will substantially contribute to the scientific literature on the development of empathy; address the knowledge gap that exists aligned with an evidence-based understanding of empathy as a construct and its associated dimensions from the perspective of HCPs and caregivers of PLWD; impact how empathy is conceptualized and taught within our curriculum and community; finally, contribute to guiding further research aligned with implementing AR as an experiential education to enhance empathy among HCPs and caregivers of PLWD.

## Conclusions

The focus group results have laid the foundation for designing and developing a platform that will be used in the later stages of our research. The results will be used to generate a plan of action for designing the AREduX prototype. The AREduX will allow end-users to build an understanding of the symptoms experienced by PLWD and will enable us to evaluate how the medium impacts empathy levels among HCPs and caregivers of PLWD.
